# Annotations

**Published:** 1893-05-20

**Authors:** 


### ANNOTATIONS.

**Death Certification and Stillborn Babies.**

BEFORE the Select Committee of the House of Commons last week Dr. R. R. Rentoul, of Liverpool, gave some startling facts on the subject of death certification. In this country alone, in one year, 15,000 stillborn children were interred in 1,300 cemetries; and of these as many as 4,500 were interred without any medical certificate whatever. It appears that in some instances cemetery authorities ask for declarations that children have been stillborn. But there are apparently no steps whatever taken to verify the accuracy of such declarations. It would seem, therefore, that a person may do to death any number of helpless infants and get them buried as stillborn without the least difficulty by merely making repeated declarations. Dr. Rentoul very properly suggests that no stillborn child shall be buried without a duly filled up certificate, signed by a registered practitioner. He suggests also that the certifying practitioner must certify that he has properly inspected the body after death, as well as attended the case during life. This, like so many other reforms, merely needs to be stated to be generally approved. We must do our utmost to enable the eye of civilisation to search into every corner and hiding place of our complicated social activity, and the hand of civilisation to reach every offender against the sacredness of human life. It is pitiful to think that there are 4,500 unhappy babies, more than 12 for every day in the year, for whom nobody can be found to say whether they are born dead, or are cruelly and inhumanly murdered by some fiend in human form as soon as they appear in our terrible world. More than 12 for every day in the year: one for every hour of the day, and nobody to care whether they are born dead or alive, unless, indeed, it be those foul monsters who await the moment of their birth in order to put a violent end to their innocent and helpless lives. This is our civilisation. Many more heartrending facts were brought forward by Dr. Rentoul. But, for pity's sake, let this modern murder of the innocents be grasped and dealt with first.

**The London's Quinquennial Appeal.**

WE never single out a particular hospital for special mention, except for special reasons. When, therefore, we put the London Hospital prominently forward, we have no desire to hide the merits or the necessities of other institutions of a like kind and of equal merit. But it is by the common consent of all medical charities that the London Hospital, once in five years, urges a special claim upon the consideration of the charitable public. The London Hospital makes this special appeal for the most imperative of all reasons—viz., because it cannot help itself. Placed where it is, it finds an overwhelming amount of medical and surgical work lying always at its doors as inevitably as the night follows upon the sunset. There are the 10,000 annual in-patients, whose cases must be dealt with immediately, or the lives of most of them would be sacrificed. They are brought to the doors of the London Hospital as naturally, and with the same faith as the sick man was to the great healer when his friends uncovered the roof because there was no entrance by the doorway, and let down the patient into the middle of the crowded room. To refuse to admit those ten thousand is nothing short of an impossibility. There they are; they have no other resource; they are struck down by mortal disease; they must be taken in. And if that be so, they must be fed, doctored, nursed, and cared for until they are better; and the money must be provided as best it can. Compared with the helplessness and the sufferings of the sick, and with the relief bestowed and the cures effected, the mere money question seems to be absolutely insignificant. But it is a stern fact for all that; and those who are responsible are sternly aware of it. A regiment of 700 patients always in residence, and of 200 doctors, nurses, and servants to take care of them, costs a tremendous sum in food and medicine alone week by week. Add to this the 112,092 out-patients—more than 2,000 for every week—and we see at a glance how vast must be the cost, and how constant and generous must be the stream of benevolence which meets that cost week by week and year by year. The London, in spite of its best efforts, accumulates a deficit year by year, and that deficit must be periodically paid off. If it be not, the hospital must sooner or later be paralysed as a charitable institution. The quinquennial appeal is designed to pay off the deficit, and to enable the hospital to do the work that falls inevitably to its share, and which either it must do, or the work must be left undone. What better arguments than these can we place before the charitable? If the London Hospital be allowed to languish, we may take it for granted that all other hospitals will soon be languishing too. It must not be allowed to languish. So far as we are concerned, we venture to urge its claims upon the public by every possible argument which can be employed.

**Vaccination: A Suggestion for M.P.'s.**

THE anti-vaccinators, like all enthusiasts, carry out their propaganda "in season and out of season." Vaccinators, who have a reasoned and intelligent faith in vaccination, do not object to the enthusiasm, or to its occasional unseasonableness. By reasoning and counter-reasoning truth is brought to light. The House of Commons has declined for the present to put an end to compulsory vaccination. But the report of the Royal Commission, which will no doubt be published within a measureable number of months, will be the signal for the closing up of the anti-vaccinators' ranks, and a determined effort to carry the position of the compulsionists. For our part, we feel that a majority must have its way. There is no reason why medical men should insist upon benefitting the country against its will. But in the meantime, we venture to suggest that the members of the House of Commons, severally and collectively, should take a little trouble to make themselves acquainted with a subject on which such momentous issues depend, before they venture, by their votes, to enact statutes which may mean death to thousands, and maiming and disfigurement to tens of thousands more. If the medical profession cannot be trusted to decide such a question as that of compulsory vaccination, on what ground is it proposed to trust the House of Commons? If a fish may not be relied upon to swim scientifically, would it be scientific to suggest that a dray-horse might do it better? We must have a little faith in one another, or reason, religion, and the whole of the business of the world will come to an end. Half-a-dozen cynical anti-vaccinators may try to believe that the thirty thousand medical men of Great Britain are made up of fools and scoundrels. But such a proposition is a little too monstrous for the man of ordinary sense and honesty to swallow. The present writer has always insisted, in this as in other well-known cases, on hearing both sides. With this object he has carefully studied anti-vaccination literature, and quite recently has read Dr. Creighton's "Jenner and Vaccination." Now, Dr. Creighton is one of the bitterest of all the anti-vaccinators, and his book is a very skilful example of special pleading. Nevertheless, the effect of reading the book was to convince the writer that Jenner and subsequent vaccinators have made out a reasonable case for vaccination, and that the anti-vaccinators, as represented by Dr. Creighton, are incompetent and untrustworthy critics. We venture to think that any educated member of Parliament who reads critically Creighton's attack on Jenner, will rise from the study of this anti-vaccination book a more than half-convinced vaccinator.

